# The Compressiometer: Toward a New Skin Tensiometer for Research and Surgical Planning

**DOI:** 10.1109/JTEHM.2021.3133485

**Published:** 2021-12-06

**Authors:** Karlijn M. J. Scheepens, Nick Marsidi, Roel. E. Genders, Tim Horeman-Franse

**Affiliations:** MechanicalMaritime and Materials Engineering DepartmentTU Delft 2860 Delft 2628 CD The Netherlands; Leiden University Medical Centre 4496 2333 ZA Leiden The Netherlands; Ziekenhuisgroep Twente 1154 7555 DL Hengelo The Netherlands; Sustainable Surgery and Translational Technology, MechanicalMaritime and Materials Engineering DepartmentTU Delft 2860 2628 CN Delft The Netherlands

**Keywords:** Incision planning, langer lines, medical device, skin tension, tensiometer, surgery

## Abstract

After surgery, around 35% of patients experience problems of excessive scarring, causing disfiguring and impaired function. An incision placed in the wrong direction causes unnecessary skin tension on the wound, resulting in increased collagen disposition and potentially hypertrophic scars. Currently, skin tension lines are used for incision planning. However, these lines are not universal and are a static representation of the skin tension that is in fact under influence of muscle action. By designing a new skin force measurement device the authors intend to make research on dynamic skin characteristics possible and to objectify incision planning and excision closure planning. The device applies a known compressive force to the skin in standardized directions and measures the displacement of the skin. This allows users to measure the skin reaction force in response to compression and to determine the optimal incision line or best wound closure direction. The device has an accuracy of 96% and a sensitivity of < 0.01 mm. It is compact, works non-invasively and standardizes measurement directions and is therefore an improvement over previously designed skin tensiometers.

## I. Introduction

Worldwide, 234 million major surgical procedures are done each year [1]. About 35% of patients undergoing surgery develop a hypertrophic scar [2]. Scars are not only an aesthetic problem causing disfigurement, they can also induce impaired function of the body part, for example due to contractures [3], [4]. The formation of hypertrophic scars is mainly related to the tension on wound edges and surrounding skin [5], [6].

**FIGURE 1.**
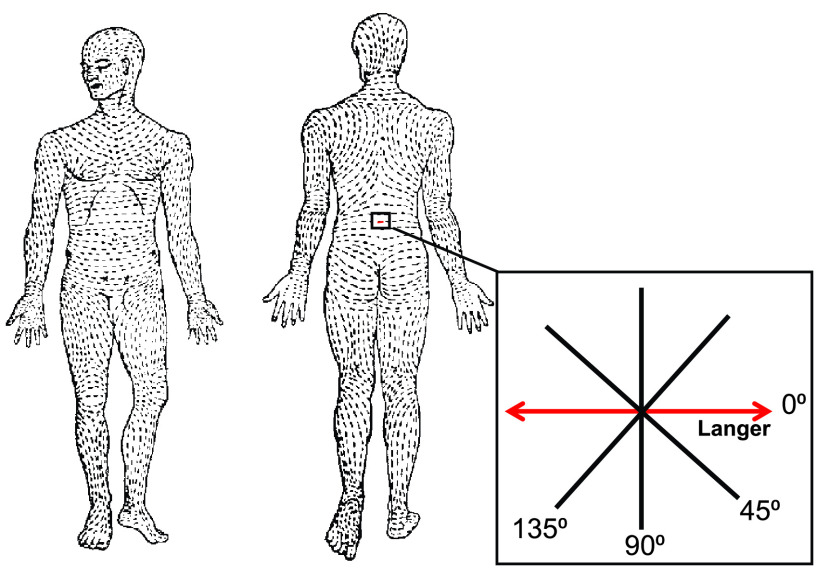
Reprinted Langer lines (skin tension lines) with measurement directions of pilot study indicated in the square.

The skin is anisotropic, which means that the material properties vary with loading direction [7]. According to Son *et al.*, the most important cause of extensive scarring reactions like hypertrophic scars is improper incision planning. An incision placed in the wrong direction causes unnecessary skin tension, pulling the wound edges apart. In order to close the wound, increased collagen deposition is needed to pull the edges together, resulting in a hypertrophic scar [8]. Currently, incision planning is done by using the skin tension lines. In 1861, anatomy professor Langer punched circular holes in cadaveric skin at different locations and observed the change of shape of the holes. Based on his observations, he drew a map of maximal skin tension lines [9], which are reprinted in Fig. 1 [10].

With microscopy he showed that these lines follow the pattern of the elastic fibers forming the skin, such as collagen [9]. It was proven that incisions parallel to the so-called Langer lines require less force to be closed [11] and due to reduced tension heal better and show less scarring [12], [13]. In elective surgery, there is often an alternative way of incision possible, besides the usual practice. In response to these findings, surgeons try to make their incisions parallel to these skin tension lines if the requirements of the intervention allow this.

However, these lines turned out not to be universal [14]. Especially in case of a non-average body posture regarding BMI or muscularity, the deviation from the Langer lines is expected to be large. Moreover, the Langer lines represent a static tension on the skin, whereas it has been found that the direction of highest tension on the skin changes due to dynamic motions as, for example, in facial expressions [15]. An alternative to the Langer lines are Kraissl’s Wrinkle lines that are revealed by pinching the skin in several directions [16]. However, the results of this method are variable, as there are differences in force applied and interpretation of the resulting lines by the surgeon. For wound closure, existing skin tension lines are even less applicable. If an excision is done, the structure of elastic skin fibers, elastin and collagen, is changed and this results in change of skin tension [17]. New research introduced the Best Excisional Skin Tension (BEST) Lines to plan wound closure after excision of skin lesions [18]. Unfortunately, these lines are still static and are not defined on all body locations. Given the mentioned drawbacks of skin tension lines, incision and excision closure planning remains difficult. As there was no alternative in clinical practice, skin tension lines were the best option up till now as quantitative measurement of skin tension was not practical nor reliable [8].

Research has been done on measuring skin tension quantitatively in-vivo, resulting in devices using various measurement principles. Skin tension measuring devices usually apply a stress to the skin and measure the resulting deformation or reaction force [19]. The stress can be applied in the form of compression, traction, suction or a combination of those options. Some of the described devices only measure the direction in which the tension is dominant [20], [21], while others also quantify the tension on average [22], [23] or in a certain direction [24]–[30]. Only the devices applying traction and/or compression have shown to be capable of providing both stress level and direction.

Although several skin tensiometer prototypes have been produced and a few are commercially available, no such device is widely used in clinical practice. Most prototypes are bulky and have cables that make them unpractical to use in the operation room [24], [27], [28], [31]. However, one exemption was found in the work of Paul *et al.* that shows the highest potential for use in the operation room, due to its compact design [25]. Nevertheless, it has a few important drawbacks. The rods applying the compression or traction are piercing the skin, making the measurements invasive and the device not ideal for incision planning on intact skin. Moreover, the directions of measurement are not standardized as the device is rotated by hand in between measurements. None of the previously described devices includes a method to standardize the directions, although this is important to derive skin tension lines in a repeatable way.

All quantitative skin tension measurements published up till now, were derived from the forearm or upper arm. On other locations, only skin tension lines have been obtained. However, knowing the tension properties on various locations and during dynamic movements could allow surgeons to adjust their surgical techniques and postoperative advices to these properties more optimally.

All in all, quantitative skin tension measurement could be useful to reduce skin scarring problems. Therefore, the goal of this research is to design a device that measures the reaction force in the skin after compression. With this device it should be possible to execute three functions:

1) Investigate the dynamic characteristics of the skin tension under compression.
2) Find the individual most optimal incision direction at the intended location.
3) Determine in which direction to close a wound after excision on locations where BEST lines are undefined.

## II. Methods and Procedures

### A. Requirements

In general, the device should be easy to handle for medical doctors. This means that a doctor can carry it in a breast pocket, that it only needs a surface with the size of the back of a hand for measurement and that it doesn’t take more than five minutes to execute the measurements on a location. Moreover, it should be safe for both patient and doctor and be built for under approximately 100 euros.

For research on skin tension dynamics, the device should be wearable during activity and must measure the moving skin continuously. In this case, the accuracy is formulated in terms of a tension instead of a direction. The accuracy should be 97%, equal to the value reached by Paul *et al.*, [25]. Independent from its use, the device must be cleanable with a removable disposable interface (e.g. stickers) between the sensor and skin.

If the device measures the most optimal direction for the incision, it is used on intact skin and that should not be damaged in any way. Coutts *et al.* showed differences in skin tension between directions with an angle of 22.5 degrees in between [26]. Therefore it should be possible to determine the principal direction with at least 22.5 degrees. To be broadly applicable to wound closure planning, the device should be able to apply a force to edges of wounds with various sizes, specified as one to ten millimetre diameter.

### B. Measurement System, Design and Fabrication

**FIGURE 2.**
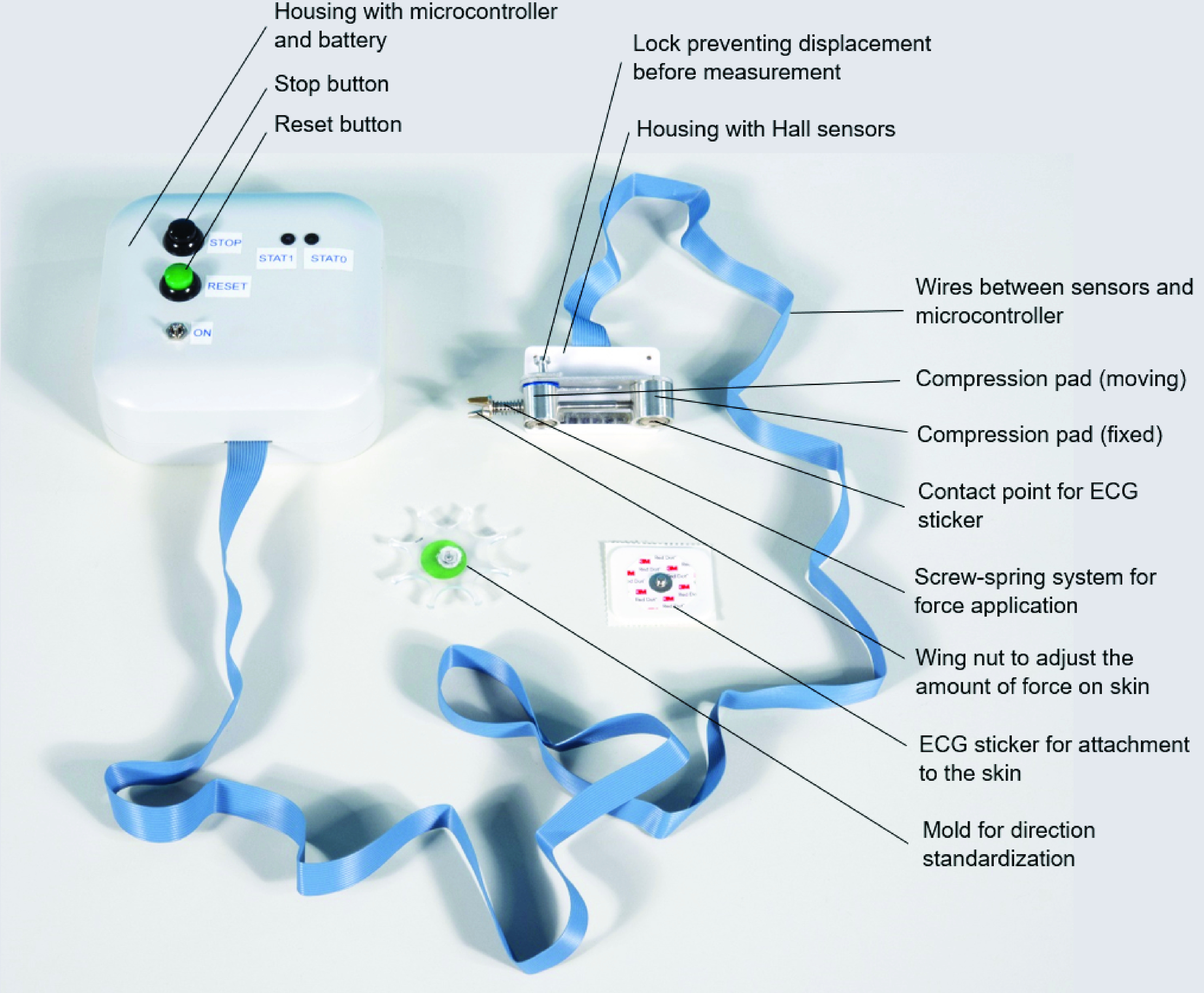
Parts of the Compressiometer.

**FIGURE 3.**
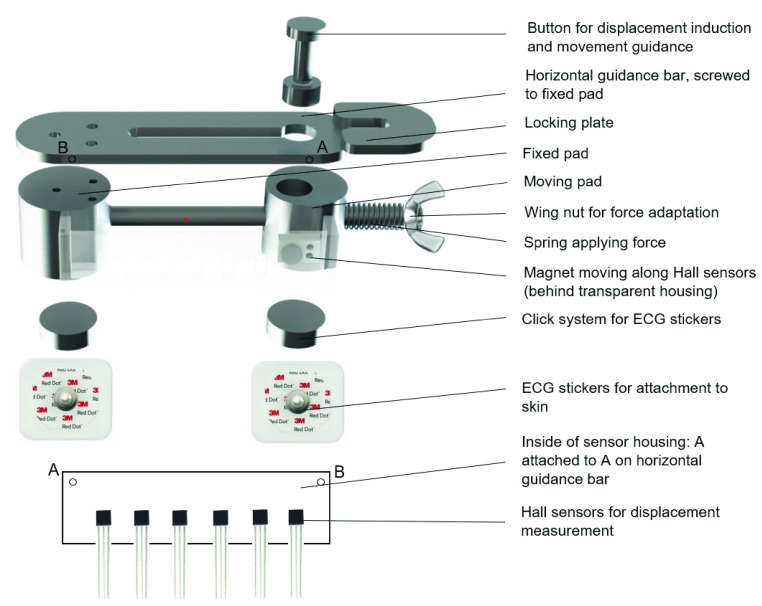
Exploded view of the mechanical parts of the device.

Based on a systematic design approach, a morphological overview was made (supplemental file A), in which logical combinations of functional components were assessed leading to one final design for the ‘Compressiometer’ skin tensiometer, as shown in Fig. 2. The device compresses the skin between two pads using a standard amount of spring force and measures the displacement. It consists of a patient side with the mechanical parts and sensors. The electronics side contains the microcontroller and battery, connected by a flat wire. The final prototype was manufactured from aluminium, stainless steel and printed ABS components. The device is attached to the skin with two pads, using commonly used electrocardiography (ECG) stickers. In starting position, the pads are 30 mm apart. A known force is applied to one of the sides by means of a spring. Before the measurement starts, displacement of all moving components is prevented by a locking system. Before the measurement is started, the electronics are turned on and the device is kept vertical with respect to the skin. If the lock is released, the pads of the device slide towards each other until an equilibrium between the spring force and skin reaction force is reached. The amount of displacement is measured by Hall sensors and read out from the Sparkfun Logomatic microcontroller as soon as this equilibrium is reached. From the displacement, the skin reaction force can be calculated. In Fig. 3, the patient side of the device is shown in exploded view. The circuit of the sensors and microcontroller with battery is included in supplemental file B.

#### 1) Force Application

A compressive force is applied to the skin using a customized force application system with a screw, a linear spring and a force scale (see Fig. 3). Although most prior research applied tensile stress to investigate skin properties [26]–[28], [32], we chose to apply skin compression as this is closest to compressive skin suturing. Compressive force was also applied by De Jong and Paul *et al.* [24], [25]. Flynn *et al.* used a device with one pad applying compression and traction at the same time in response to moving [31]. In addition, Ogawa *et al.* showed that large compressive forces needed for wound closure, lead to high tension on the wound edges after closure, which is disadvantageous for wound healing [6]. These studies indicate that the compressive force is directly related to the skin healing characteristics we want to improve with this device. Capek *et al.* investigated the forces needed to close elliptical wounds with a size in the range of $36\times 5$ to $45\times 14$ mm. The range of forces found in this study was 1.1 – 3.2 N. The forces needed were related to the natural tension of the skin and to the size of the elliptical wound [33]. To mimic wound closure, a force of 3 N was chosen. From skin tension studies performed on intact skin, the amount of compression of the skin in response to an applied force of 3 N has been estimated to be maximally 30 mm [24], [28], [29]. The distance between the pads and the unloaded length of the spring have been adjusted to this.

#### 2) Compression Pads

When the spring lengthens, the skin between two round aluminium pads is compressed. The axis with spring around it goes through the sliding compression pad and is screwed into the fixed pad.

The force is transferred to the skin by modified ECG stickers (3M, (Maplewood, Minnesota, United States) ‘red dot’ type 2560) that are clicked to the device. Two ECG electrodes are glued into recesses on the bottom of the pads to facilitate this. The fixed pad is screwed to the horizontal guidance bar. During force application, displacement is prevented by a locking system.

#### 3) Sensors

To reach the measurement range of 30 mm and achieve low friction, an array of evenly spaced Hall sensors was created and a calibration logarithm was written to convert their outputs into a reliable displacement measurement. The sensors are attached to the fixed compression pad and the magnet is attached to the moving pad, keeping the vertical distance between the magnet and each of the sensors constant during compression. The fragile Hall sensors are covered in a 3D printed housing to fixate their position, protect them from impacts and to increase the electrical safety of the device. The magnet moves through an opening in the housing. How the device with Hall sensors is calibrated, is explained in supplemental file C.

#### 4) Direction Standardization

To standardize the directions in which the stickers are attached to the skin and thus in which the device will measure, a plexiglass mold is used. As different skin tension line mappings are running perpendicularly at some locations, it was chosen to compare force measurements around a full circle. To limit the needed time, measurements are done in four directions during the pilot study presented here. Therefore, the mold has eight cut-outs in which small stripes need to be marked before measurement. During measurement, the stickers are consecutively placed on top of two opposite marks. After one measurement, these stickers are removed and the next two can be applied.

### C. Validation Tests

For the three intended functions of the device – incision direction, wound closure direction and dynamic skin research–different validation tests are relevant. To detect the most optimal incision direction or wound closure direction, the accuracy is the angle in degrees between the directions of highest force that can result from a measurement. This value can be adjusted to user preferences, as it is determined by the type of mold used and the measurement protocol. Minimizing this value is limited by the steps of direction standardization.

For the research purpose, however, it’s important to compare the quantified tension values between locations and anatomical positions. Therefore, the accuracy needs to be formulated as a percentage, related to the deviation of repeated tension measurements. For this accuracy test, a surface with homogeneous stiffness was used. Eleven measurements were performed on a piece of foam with press stud stickers glued to the surface to avoid slipping. The standard measurement procedure was followed and the two lowest sensor outputs were used for the accuracy calculation.

For all three functions, it is relevant to determine the sensitivity of the device. As four force measurements are compared to find the most optimal incision or excision closure direction, it is important to know how large the displacement differences between two possible incision directions need to be. To measure the sensitivity, the calibration setup with a caliper was used. The output of the sensors is analyzed after each 0.01 mm displacement over the range.00 to.10. This was done at 5, 15 and 25 mm distance between the pads.

### D. Pilot Study

**FIGURE 4.**
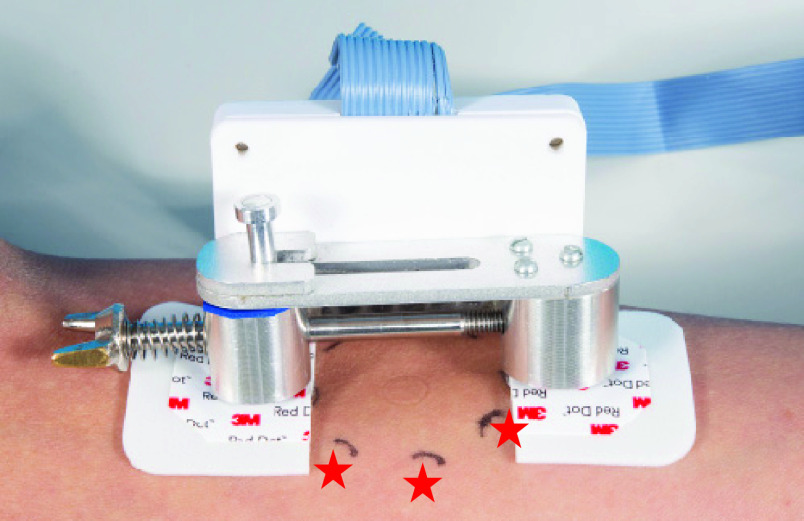
Measurement setup during pilot study. Marks (*) indicate were the pads are placed during a single trial. The indications are made on the skin with a mold and marker for direction standardization.

**FIGURE 5.**
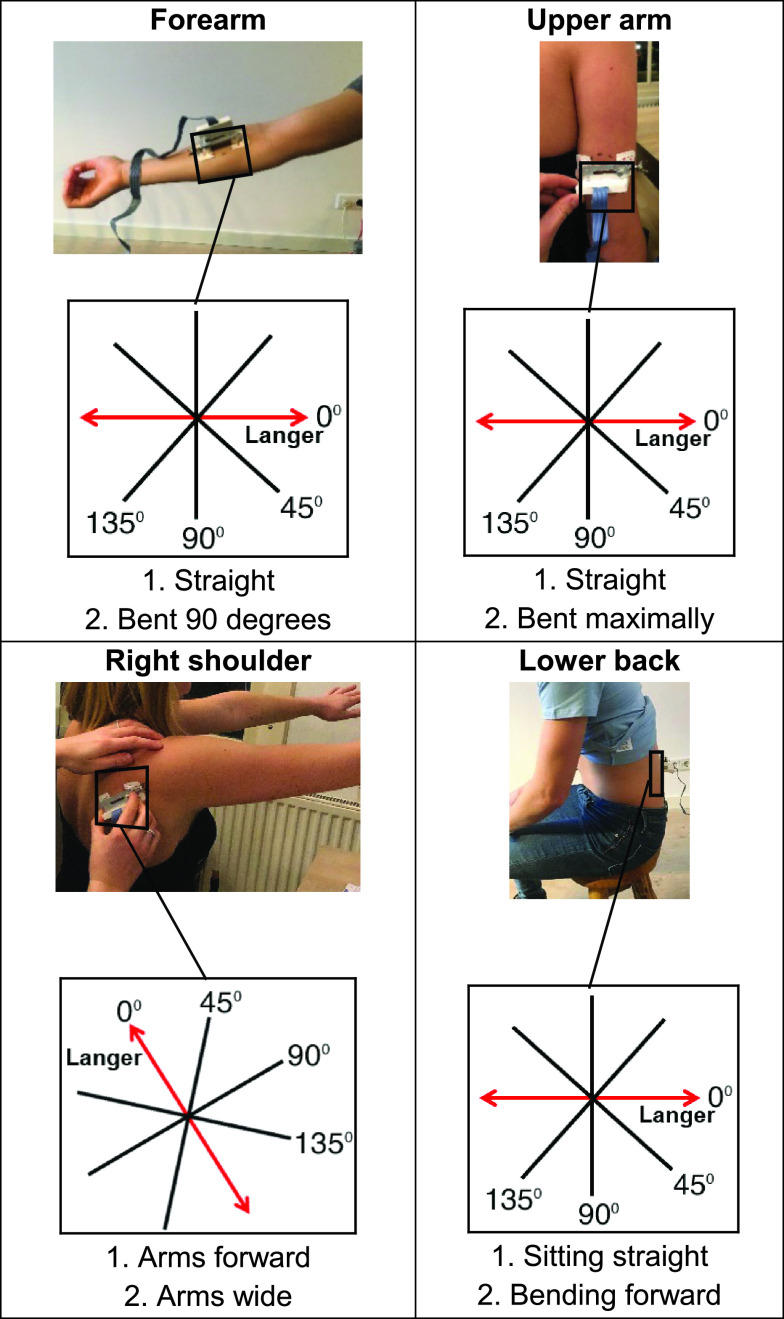
Locations of measurements and anatomical positions that were researched during the pilot study.

To start the skin dynamics research and to get an idea of the optimal incision direction in different persons on different locations, a pilot study was performed with 10 healthy participants (22-34 years of age, 9 female, 1 male). Healthy participants between 18 and 40 years old were recruited via social media to participate in this pilot study. A participant was determined healthy when that person was not familiar with a skin disease at the measurement sites nor had a systemic disease that possibly could influence the skin. Exclusion criteria were: wounds or damaged skin at one of the four measurement locations, contact allergy for aluminium or ‘red dot’ ECG stickers. Skin tension was measured at four locations (volar forearm, posterior upper arm, right shoulder blade and lower back) and for each location, two anatomical positions were adopted to get an idea of the dynamic properties of the skin. The arm locations were selected in order to be able to compare to prior research [24], [26]–[28], [30], [32], the other two were considered interesting based on the range of motion around it. The setup on forearm skin is shown in Fig. 4. All included locations and anatomical positions are shown in Fig. 5. Measurements were done in four directions of which direction Number 1 was following the Langer line at that location and Numbers 2–4 were rotated 45, 90, and 135 degrees in clockwise direction, respectively, as visualized per location in Fig. 5. The Langer line direction used in the pilot study was copied from the original study of professor Langer, published again in the British Journal of Plastic Surgery in 1978 [9]. This pilot study was approved by the METC-LDD. The study was registered in the Dutch Trial Register (NTR) with the identification number NL8476. A student T-test was done to indicate statistical differences between both configurations of the body parts. A p-value < 0.05 was considered to be statistically different.

This pilot will be expanded later to a clinical study with 30 participants. The mean skin force over the four measurement directions was compared between the two anatomical positions per location. Furthermore, the most optimal incision line - the direction of highest tension - was determined for each participant in every situation.

## III. Results

### A. Validation

**FIGURE 6.**
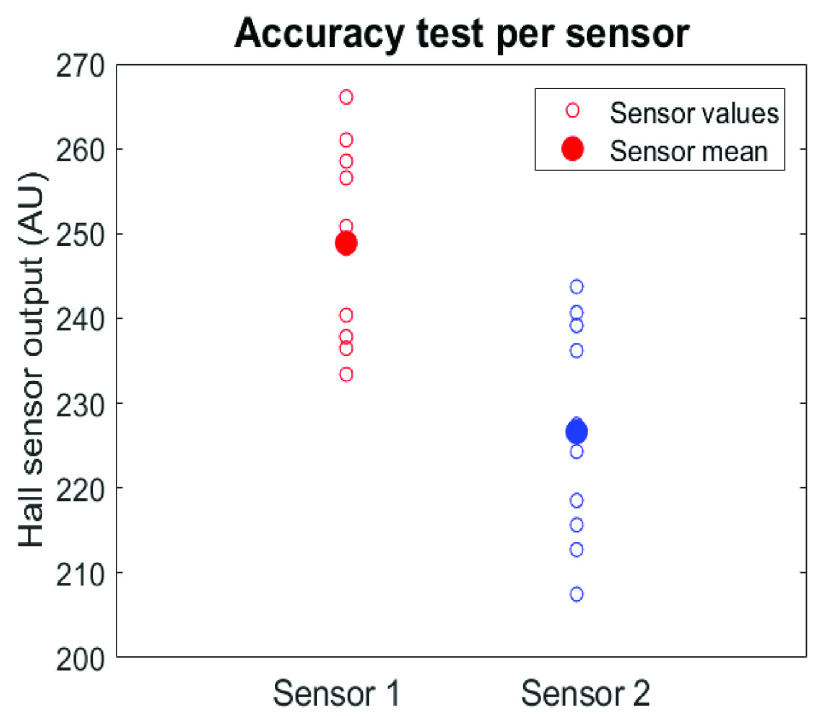
Results of 11 repeated measurements for accuracy calculation. The two groups correspond to two of six Hall sensor outputs that represent the moving pad position of the device with fixed range.

**FIGURE 7.**
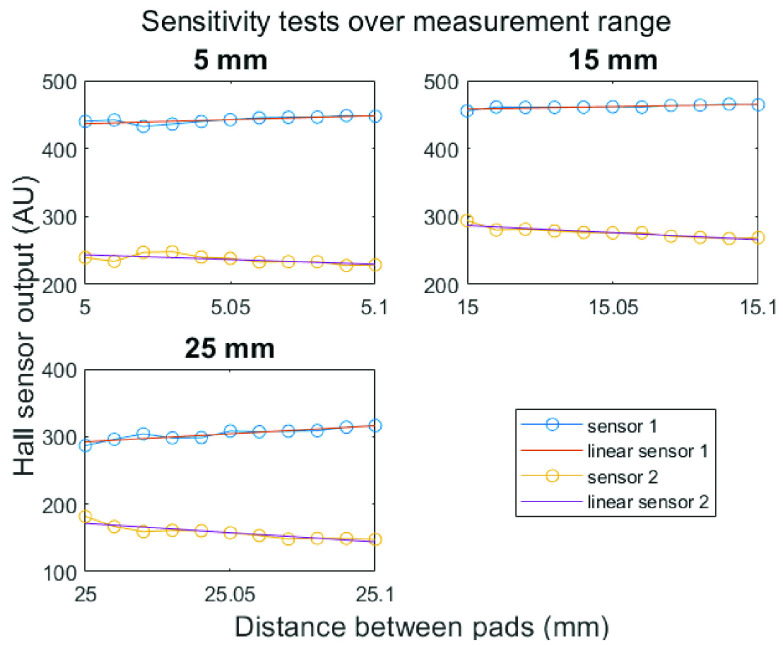
Sensitivity tests over small measurement range. The two dotted lines in each graph correspond to the output data from the two out of six Hall sensors that measured the displacement of the pad of the device.

Fig. 6 shows the quantitative skin force accuracy measurements, given by the repeated measurements and the dispersion around the mean for the relevant sensors R and RR. Accuracy R = (1 – 0,0355) $^ \ast \,\,100=96$,4%. Accuracy RR = (1 – 0,0437) $^\ast \,\,100=95$,6%. Mean accuracy = accuracy device $=96.0$%. However, the accuracy of incision and wound closure direction measurement is dependent on the angle between two tension measurements. This tension direction accuracy is estimated to be 10°. Any direction can be chosen for measurement if the mold is adapted to this, but small inaccuracy is introduced by the use of markers for direction standardization. The graphs in Fig. 7 show the sensitivity test of the two relevant sensors per location. It’s clear that the output changes after every step of 0.01 mm. Sometimes a small bump is seen in the graph, which is probably the result of inaccuracies in positioning of the pad. The trend lines show that the relevant outputs slightly decrease or increase. Therefore, the sensitivity is lower than 0.01 mm over the whole range of the device.

### B. Pilot Study

In this small study the skin force measurements are compared between directions, locations and anatomical positions. The direction of highest skin force is indicated per participant for every situation in Table 1. On the forearm and on the shoulder, the direction of highest tension changed in most cases as soon as the position was changed.

**TABLE 1:** Difference Between Measured Direction of Highest Reaction Force and Langer Line (Degrees)

Participant	Gender (Male/Female)	Forearm		Upper arm		Shoulder		Lower back	
		Straight	Bent 90 degrees	Straight	Max bent	Arms forward	Arms sideways	Sitting upright	Bending forward
1	F	0	90	135	135	0	0	90	90
2	F	45	135	90	90	135	45	90	135
3	F	0	135	135	135	0	0	90	90
4	F	0	135	90	135	0	45	90	90
5	F	45	135	135	135	135	0	135	90
6	F	0	90	90	90	0	0	90	90
7	F	135	90	90	135	0	45	135	45
8	M	0	90	135	135	0	45	90	90
9	F	0	135	90	90	135	0	90	90
10	F	0	135	90	135	0	45	90	90

**FIGURE 8.**
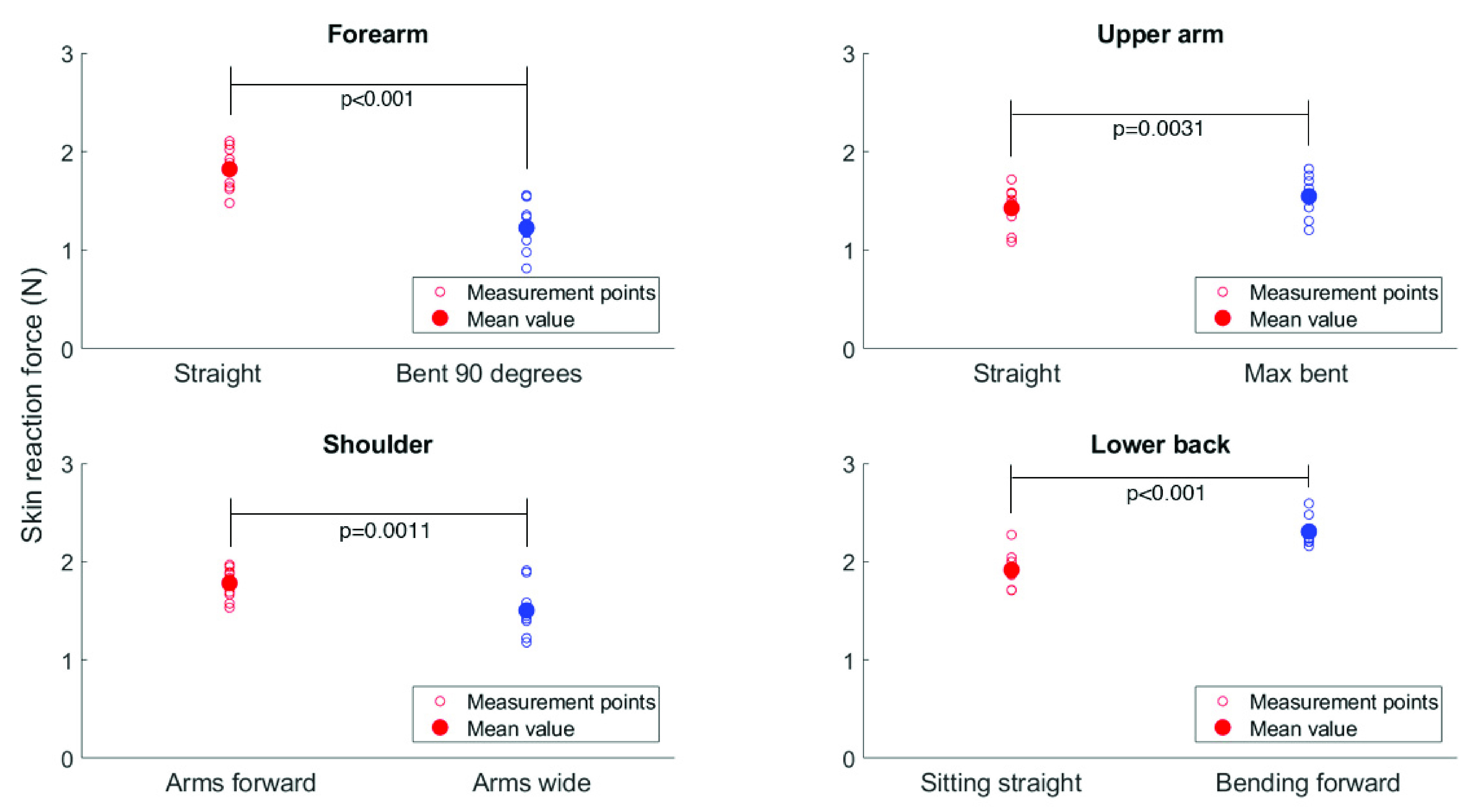
Results of pilot study on ten participants. Skin reaction force measured on four locations, each in two anatomical positions. Each measurement point is the mean over four measurement directions. The p-values indicating significant differences are shown.

In every column, indicating one specific measurement situation, two different directions appear as the most optimal incision line. Moreover, Fig. 8 shows the measured skin force values per location in a plot. The two anatomical positions are depicted next to each other. For each location, a significant difference was found between both body configurations, with p-values of < 0.001 (forearm), 0.0031 (upper arm), 0.0011 (shoulder) and < 0.001 (lower back).

## IV. Discussion

The results indicate that the study aim is reached and the designed Compressiometer can potentially help a surgeon plan surgical incisions. The device was successfully used for the three defined functions:

1) Investigate the dynamic characteristics of skin tension under compression that is currently not anticipated on in surgery or aftercare.
2) Find the individual most optimal incision direction at the intended location.
3) Determine in which direction to close a wound after excision on locations where BEST lines are undefined.

The device applies compression to the skin area of interest and measures the resulting displacement under loading. From the known applied compressive force and displacement, the reaction force in opposite direction can be derived. The measurements are done in standardized directions to derive the direction of highest reaction force with an accuracy of 10%.

### A. Requirements

The general device requirements related to size, safety, measurement time and costs were met with the current Compressiometer design. However, the current prototype design is not yet able to fulfil all three functions in an optimal way.

Although not all requirements are met yet, this device is ready to take the first step in skin tension dynamics research, its first function. The accuracy of the displacement measurement and thus skin reaction force was found to be 96%, slightly lower than the 97% reported by Paul *et al.* [25]. Continuous measurements are possible with the Hall sensors used, but the calibration script that results in the final force measurement cannot function continuously. In order to improve this, the microcontroller should be changed and reprogrammed. To do continuous measurements during activity, it would be advantageous to include the electronics in a smaller housing and to create a customized type of sticker that allows more robust fixation.

The second goal was to find the optimal incision direction prior to an operation. In case of an average body posture, the Langer lines might suffice, but especially in case of non-average BMI or muscularity, this device could be the solution to obtain optimal skin healing. For practical reasons, a mold with four directions 45 degrees apart was chosen to do the pilot study. If this mold is adjusted, an accuracy of 10 degrees can be reached. As the ECG stickers make the device non-invasive and the contact surface device can be cleaned with alcohol, all requirements for the incision function were met.

Finally, the device was aimed to indicate the wound closure direction after excision, as the BEST lines created for this purpose are on some body locations, such as facial skin, hands and feet, undefined. The current version of the device is not ready for this, as the distance between the pads is not adaptable to the wound size. By rotating the wing nut, the amount of force applied can be adapted. To be applicable during surgery, the disposable interface between the sensor and skin should be extended to cover all non-sterile parts. The stickers used are sterile and can be directly attached to the skin.

### B. Pilot Study

The pilot study showed that there is a clear relation between the orientation of the body parts and measured reaction force. Moreover, the individual direction of the highest reaction force is not constant and can depend on individual characteristics as well as the orientation. The values found were in the range of 1 - 2 N. The T-tests indicate a statistical difference for the two anatomical of the forearm (p $= 8.5^\ast 10^{-7}$), upper arm (p = 0.0031), shoulder (p = 0.0011) and lower back (p $= 3.7^\ast 10^{-6}$). The arm locations could be compared to previous research. As the experimental methods differ highly between studies in literature, it is not surprising that differences in outcomes occur. Mofid *et al.* compared the mean tension on the volar forearm between participants by applying traction and found values between 1.25 and 1.44 N [29], which is close to our own findings. Flynn uses a micro-robot that is attached to the skin with one pad and thus combines traction and compression and concluded that the reaction force of the skin at maximum displacement was around 1.6 N for both the volar forearm and the posterior upper arm [30]. Coutts uses traction and measured higher force at the same location, between 2.3 and 3.5 N [26]. A few authors find lower tension values. Gahagnon *et al.* apply traction and report 0.23 - 1.47 N on the forearm [28]. De Jong shows results of 0.4 - 0.6 N after applying a compressive force of the skin at the same location [24]. Boyer finds a forearm force of 0.05 – 0.3 N [27] after skin traction.

From the pilot results it is expected that the skin tension levels indeed change when a person moves. Moreover, interindividual variation was detected in the direction of highest reaction force, which is the most optimal incision line. If these findings are confirmed in a larger clinical study, this would mean that the surgical planning based on skin tension mappings is often suboptimal.

It is expected that this device is especially useful for more complicated surgical planning situations, where the optimal approach is not perfectly clear beforehand due to personal factors, pathology or affected skin tissue. In a commentary in the Dermatology, adaptation of breast incision lines to breast size and shape is proposed, as tension on the skin is highly related to these factors [34]. In case of extraordinary BMI or muscularity, similar advice could be derived from individual measurement using the Compressiometer. By using the results of this research, surgeons can become more aware of the importance of the incision direction for wound closure planning and the possibility to optimize their approach.

### C. Limitations

The current version of the Compressiometer is still a prototype. For frequent use, the device needs to be more robust. In the new design, the part getting in contact with the skin must be separated from the electronical parts by some disposable plastic for sterile use.

As regards measurement area, the current device needs a minimal flat surface of around 3000 mm^2^ for operation which equals a circle with a 3 cm radius. To make the device more applicable for smaller skin surfaces like forehead or scalp, the interface pads attached to the skin need to be optimized further. Moreover, the influence of surface curves along the displacement trajectory on the measurement should be tested. It is not yet possible to read out the results on a screen right after the measurements. Adding this feature would save energy and time in the operation room and enlarge the chance of implementation in the clinic.

Another drawback of the device is the need for markers in order to standardize the measurement directions. This decreases the accuracy of the directions, as two separate actions are needed to position the stickers. Moreover, markers could induce allergic reactions. In the ideal case, the device would be self-adhesive. However, direct fixation using suction influences the skin tension. Stickers are therefore the best alternative, although stickers small enough to be attached around the mold could not be found. To be independent of markers, a customized sticker has to be produced with a press stud of the size of the compression pads and a layer of foam and glue underneath.

The results obtained with the compression method are considered to be more clinically relevant than with traction, because this method mimics compressive wound closure. However, the bulking of the skin between the pads could limit the displacement and influence the results.

### D. Future Work

To determine the effect of incision tension on post-operative scar development, more detailed clinical long term studies are needed. Our next step is to expand this pilot study into a larger clinical study on dynamic skin tension properties in healthy participants. How these dynamic characteristics can exactly be anticipated on, is to be found out in future research. For example, it could be investigated whether the incision direction should be most optimally chosen according to the direction of highest tension in an anatomical position that is common during daily life or sports where larger tensions play a role. Moreover, after wound closure, certain bandages or strips could be tested that support the scar during movement. Revision of the prototype is needed to execute the second function of wound closure planning, which asks for immediate result presentation and the possibility of adaptation to wound size.

## V. Conclusion

In this Early-Clinical Research, a Compressiometer device is presented which may help surgeons optimize surgical planning and possibly achieve better results. The non-invasive Compressiometer has shown to be accurate and compact enough to measure differences in the skin reaction force to compression on relevant locations on the body and between different participants. Although there is room for improvements, the Compressiometer can be used for further investigation of the skin force variations induced by relative body part movements. Although the effect on scarring is not yet proven, the pilot data indicate that it would be beneficial to integrate objective skin force measurements in surgical planning to ensure proper wound healing, especially in more complicated situations.
